# An Efficient Early-breaking-estimation and Tree-splitting Missing RFID Tag Identification Protocol

**DOI:** 10.3390/s23239318

**Published:** 2023-11-21

**Authors:** Mingqiu Fan, Lijuan Zhang, Lei Lei, Chunni Yu

**Affiliations:** College of Electronic and Information Engineering, Nanjing University of Aeronautics and Astronautics, Nanjing 211106, China; fanmingqiu@foxmail.com (M.F.); leilei@nuaa.edu.cn (L.L.); yuchunni0228@163.com (C.Y.)

**Keywords:** RFID, IoT, missing tag identification, unknown tag, tree-splitting, tag number estimation

## Abstract

Retailers grapple with inventory losses primarily due to missing items, prompting the need for efficient missing tag identification methods in large-scale RFID systems. Among them, few works considered the effect of unexpected unknown tags on the missing tag identification process. With the presence of unknown tags, some missing tags may be falsely identified as present. Thus, the system’s reliability is hardly guaranteed. To resolve these challenges, we propose an efficient early-breaking-estimation and tree-splitting-based missing tag identification (ETMTI) protocol for large-scale RFID systems. ETMTI employs innovative early-breaking-estimation and deactivation methods to swiftly handle unknown tags. Subsequently, a tree-splitting-based missing tag identification method is proposed, employing a *B*-ary splitting tree, to rapidly identify missing tags. Additionally, a bit-tracking response strategy is implemented to reduce processing time. Theoretical analysis is conducted to determine optimal parameters for ETMTI. Simulation results illustrate that our proposed ETMTI protocol significantly outperforms benchmark methods, offering a shorter processing time and a lower false negative rate.

## 1. Introduction

Recently, radio frequency identification (RFID) has been widely applied in many domains, such as logistics, manufacturing, the pharmaceutical industry, and so on [1,2]. As one of the key perception technologies that enable Internet of Things (IoT) networks [3], RFID exhibits many advantages, including non-contact, non-visual reading, strong anti-interference ability, high reliability, and capablility of working in harsh environments etc. According to a study conducted by the National Retail Federation [4], retailers suffered USD 94.5 billion in 2021 due to shoplifting, inventory loss, internal theft, management errors, supplier fraud, and other reasons. Missing items have become the main cause of loss for retailers in inventory management. In these applications, readers are used to monitor tags in stock frequently for goods management and inventory.

To effectively identify the missing items, many missing tag identification protocols, including probabilistic and deterministic ones, are proposed. On the one hand, probabilistic protocols implement lightweight operations to detect the missing tag event with predefined reliability [5,6,7]. These works usually take a short time to discover a missing tag event, but they cannot provide ID information of the missing tags. On the other hand, deterministic protocols give ID information of missing tags [8,9,10,11,12]. Making use of the hash mapping method, these protocols assign known tags to different slots and identify missing tags by checking whether there is a tag response in the expected singleton slot. If no response is detected, the corresponding tag is missing. Otherwise, it is a present one. To further improve the identification efficiency, some recent works considered to use bit-tracking technology, such as the pair-wise collision-resolving missing tag identification (PCMTI) protocol [11] and the collision-resolving-based missing tag identification (CRMTI) protocol [12]. However, these works assumed that all tags within range are known to the reader without considering any unexpected unknown tags.

In practical scenarios, some unknown tags may present and affect the identification of missing tags. With the presence of unknown tags, a missing tag may be misidentified as a present one if the unknown tag is assigned to the expected singleton slot and replies a 1-bit short message to the reader. In the literature, a few works considered the effect of unknown tags and tried to deactivate them, such as the two-phased bloom filter-based missing tag detection (BMTD) protocol [6] and the efficient and reliable missing tag identification (ERMI) protocol [13]. In general, existing missing tag identification protocols have the following limitations:

- Since the reader has no prior information about unknown tags, an efficient unknown tag number estimation method is of great importance to guarantee the required reliability. For time-saving consideration, existing works either lack the estimation process or only provide a rough estimation that the required reliability is not always guaranteed;
- Existing works implement Aloha-based strategies to identify missing tags. In each frame, unidentified tags are randomly assigned to slots with hash mapping. None of them considered making use of information in the preceding frames. The slot information is not fully used and the time efficiency needs further improvement;
- In previous works, tag replies to the reader with a one-bit short response. To reduce the time cost, several works considered using customized responses with the help of bit-tracking technology. However, there still exist many short response slots that lower the time efficiency.

In this work, an efficient early-breaking-estimation and tree-splitting-based missing tag identification (ETMTI) protocol is proposed for large-scale RFID systems. In ETMTI, two new methods are developed to enhance the unknown tag deactivation and missing tag identification process, respectively. The major contributions of this work are in four folds as in the following.

(1) A new early-breaking-estimation-based unknown tag deactivation (EBUD) method is developed to estimate the number of unknown tags and deactivate them within a short time. The early-breaking factor is chosen to balance time cost and estimation accuracy, and the number of frames is determined to guarantee the required reliability;
(2) A new tree-splitting-based missing tag identification (TSMTI) method is designed to effectively identify missing tags. In TSMTI, the *B*-ary splitting tree method is developed to accelerate the identification process. The optimal frame factor and branch number in TSMTI are derived theoretically to minimize the execution time;
(3) A bit-tracking response strategy that allows simultaneous replies of multiple tags is developed to accelerate the identification process. With customized tag responses, the reader can identify multiple tags in one slot, which greatly reduces identification time.
(4) Theoretical analysis is conducted to optimize the parameter settings and derive the expressions of time cost in each phase. Numerous simulation results are presented to demonstrate the effectiveness of ETMTI. Compared with existing benchmark works, ETMTI takes a shorter identification time and a lower false negative rate to identify missing tags.

The remainder of this work is organized as follows: Section 2 reviews the most related works on missing tag identification. Section 3 gives the system model of this work. In Section 4, the proposed ETMTI protocol is described in detail. Then, theoretical analysis is conducted in Section 5. Simulation results are presented in Section 6. Finally, some concluding remarks are made in Section 7.

## 2. Related Works

In this section, we first introduce the traditional missing tag identification protocols with only known tags. Next, the related works that deal with unknown tags are reviewed.

### 2.1. Missing Tag Identification with Only Known Tags

In the last decade, many missing tag identification protocols have been proposed to specify the ID information of missing tags from the known ones. Li et al. first proposed the two-phased protocol (TPP) and two-hush protocol (THP) [8]. Next, Liu et al. proposed a multi-hashing-based missing tag identification (MMTI) protocol to improve the utilization of each frame [9]. With multiple hash assignments in MMTI, many expected empty or collision slots are changed into expected singleton slots so that more tags can be identified in a frame. Making use of multiple hash seeds, the slot-filter-based missing tag identification (SFMTI) protocol [10] reconciles expected collision slots with two or three tags into singleton slots to further improve the utilization of a frame. Later on, some similar protocols that make use of the reconcilable collision slots are proposed, such as the coarse-grained inventory list-based stocktaking protocol [14] and the collision reconciliation and data compression algorithm [15].

Considering the requirements of practical applications, Chen et al. proposed an improved vector-based missing key tag identification (iVEKI) protocol [16] to deactivate ordinary tags and identify missing key tags separately. Thus, the missing more valuable key tags can be identified more efficiently. Considering privacy-leakage prevention, Wang et al. made use of the group-based and collision-reconciled protocols to identify missing tags in blocker-enabled systems [17]. In [18], Yu et al. proposed the point-to-multipoint (P2M) and collision-free point-to-point (P2P) protocols to reduce communication costs. However, most of these works concentrate on improving frame utilization with the help of either multiple hash assignments or collision reconciliation strategies. Much useful information is wasted.

In recent research, a few missing tag identification protocols are considered to use bit-tracking technology. With Manchester encoding, the reader is capable of detecting the positions of colliding bits in the received collision message and retrieving useful information in the collision slot. Actually, bit-tracking has been widely applied in many tag anti-collision protocols, such as the *M*-ary collision tree protocol [19], efficient bit-detecting protocol [20], modified dual prefixes matching mechanism [21] and so on. For missing tags, PCMTI verifies the presence of two tags in each slot with the help of bit-tracking [11]. To further improve the identification efficiency, CRMTI takes advantage of both bit-tracking and collision-resolving technologies to allow customized tag responses in the reconcilable collision slots [12]. These strategies can reduce time costs to some extent, but they did not make full use of the bit-tracking technology.

### 2.2. Missing Tag Identification with Unknown Tags

Many works assume that the reader knows the ID information of all present tags within the reading range, which is unrealistic in most applications. In the literature, Shahzad et al. took the first step to consider the effect of unknown tags and proposed two RFID monitoring protocols with unexpected tags (RUN) [5], i.e., RUN${}_{D}$ and RUN${}_{I}$ for probabilistic and deterministic missing tag identifications, respectively. In their work, multiple frames with different seeds are executed to reduce the effect of unknown tags, and the number of unknown tags is estimated from the executed frames and used to optimize the frame parameters to reduce time and cost. Although RUN did not take any additional frames for estimation, the execution of all slots in each frame takes a long time. In [22], Xie et al. proposed a fast continuous scanning (FCS) protocol that uses multiple categories filter to detect unknown tags and skip the non-singleton slots to improve the identification efficiency.

To further reduce the effect of unknown tags, Chen et al. proposed two ERMI protocols [13] and separated the process into unknown tag deactivation and missing tag identification phases. In the first phase, the reader estimates the number of unknown tags and deactivates them. With the estimated tag number and predefined reliability, the frame parameters are optimized to minimize the execution time. In the second phase, the traditional hash assignment method is used for missing tag identification. However, the required reliability of ERMI is not always guaranteed, especially when the number of unknown tags is large. Similarly, Yu et al. introduced the BMTD protocol to deactivate unexpected unknown tags and then to detect tag missing events [6]. Following up, a compressed filter-based BMTD (CBMTD) protocol is proposed to further reduce the time cost [7]. Wang et al. also proposed a near-optimal protocol (OPT-G) [23] to notify the group ID of known tags in the presence of unexpected unknown tags.

Recently, some unknown tag number estimation protocols have been proposed. In [24], Xiao et al. studied the churn estimation problem in dynamic RFID systems and proposed three churn estimators to estimate the numbers of missing, present, and unknown tags, separately. They used the state changes caused by missing and unknown tags to estimate the number of dynamic tags, but the slots with both missing and unknown tags were wasted. In [25], Liu et al. proposed a simultaneous estimation of the blocked tag size and the unknown tag size (SEBU) protocol to facilitate the identification of blocked RFID tags. Xi et al. implemented single-slot count (SCT) and time slot reuse (TSR) strategies in SSR (SCT + TSR) protocol to estimate the numbers of missing and unknown tags simultaneously [26]. Considering unreliable channels, Wang et al. proposed a cardinality estimation scheme (CEUT) to estimate the number of unknown tags in the presence of known tags [27]. However, these works focus on increasing the estimation accuracy of unknown tag numbers and the time cost is high.

Moreover, some special strategies are introduced to mitigate the effect of unknown tags. In [28], Wang et al. proposed an order-based missing tag identification (OMTI) protocol to dynamically assign each tag an exclusive slot. With offline serialization and online identification, the effect of unknown tags is reduced. In [29], Chen et al. presented an efficient and accurate protocol to identify missing tags in high dynamic RFID systems. They combined the reply slot location and reply bits of tags for simultaneous missing tag identification and unknown tag filtering. Some unknown tag identification protocols are proposed to separate known and unknown tags [30,31]. In general, existing works take some strategies to reduce the effect of unknown tags, whereas they usually take the basic hash assignment method to identify missing tags which takes a long time to meet the high-reliability requirement.

## 3. System Model

**Figure 1:**
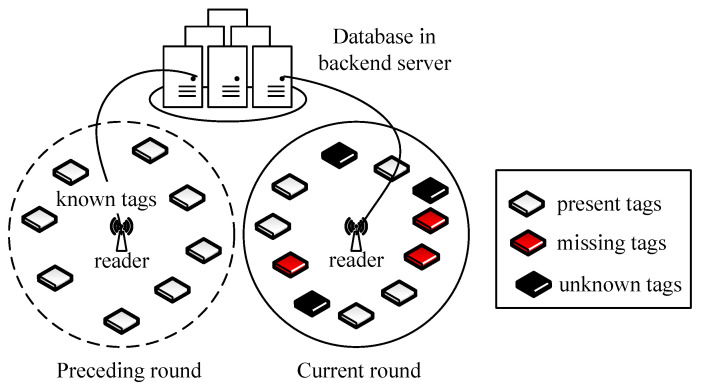
System model of a large-scale RFID system with both known and unknown tags. Note that the ID information of known tags is stored in the backend database, and the reader has no prior information about unknown tags.

This work considers a typical large-scale RFID system with a reader, a backend server, and numerous tags as in Figure 1. Tags are attached to objects for ease of identification, classification, sorting, and other inventory management. For simplicity, each object is assumed to have one tag and is represented by the corresponding tag ID. The reader is in charge of monitoring all tags within its reading range and uploads the collected ID information to the database in the backend server. Readers can also retrieve information on tags stored in the database via a high-speed channel. The backend server has powerful communication, computation, and storage capabilities that can effectively assist the reader in monitoring tags. Each tag has a unique ID and is capable of simple computation operations as in [12,13], such as random number generation, lightweight hash function, modulus operation, and so on. For the implementation of the identification protocol in practical systems, the standard of EPC GEN2 [32] provides powerful tools and standardized solutions for item identification, tracking, and management [33,34], using a commercially available RFID system to realize their identification protocols. These efforts are the foundation for the feasibility of the protocol we proposed in real-world scenarios.

With stock management, the ID and other information of new tags are collected and recorded in the backend database with traditional tag anti-collision protocols in warehouse entry. In the system, the set of tags may dynamically change because of management faults or theft. For example, some tags may be taken to the wrong zone and newly appear in the reader’s reading range; some may be stolen or mistakenly moved out of the reading range. Therefore, the reader has to frequently monitor all tags within range to identify missing ones as soon as possible.

To efficiently identify missing tags, the reader verifies the state of each tag by comparing the collected tag response with the backend database. Since the reader can retrieve all tags’ ID information from the backend database, we denote the tags stored in the database by *known tags*. A *reading round* is referred to as the process in which the reader verifies the states of all known tags. As is shown in Figure 1, if a known tag is still within the reading range in the current round, the tag is referred to as *present tag*; otherwise, it is a *missing tag*. If a tag newly appears in the reading range, i.e., there is no information in the database, it is an *unknown tag*.

We denote the numbers of known and unknown tags by K and U, respectively. The number of missing tags is represented by M. Affected by the presence of unexpected unknown tags, a missing tag may be falsely identified as present. Let $M_{fls}$ indicate the number of falsely identified missing tags. We definethe false negative rate $\nabla_{fn}$ be the number of falsely identified missing tags to the total number of missing tags. Given a required reliability α, the reader has to identify all missing tags in M and the following inequality should be guaranteed, i.e.,

(1) $$\begin{array}{c} \nabla_{fn}=\frac{M_{fls}}{M}<1-\alpha . \end{array}$$

The main objective of this work is to reduce time cost and false negative rate in missing tag identification with the presence of unknown tags in large-scale RFID systems.

## 4. Proposed ETMTI Protocol

**Figure 2:**
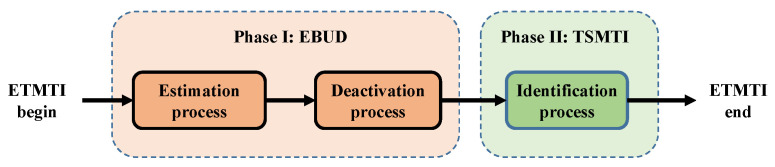
Schematic of ETMTI: (1) in Phase I, the reader estimates the number of unknown tags and deactivates them; (2) in Phase II, the reader identifies missing tags with the tree-splitting method.

In this section, we describe the proposed ETMTI protocol in detail. The identification process of ETMTI consists of two phases, i.e., unknown tag deactivation, and missing tag identification phases. As is illustrated in Figure 2, a new early-breaking-estimation-based unknown tag deactivation (EBUD) method is developed in Phase I to effectively estimate the number of unknown tags and deactivate them. With EBUD, most unknown tags can be deactivated in a very short time. In Phase II, a new tree-splitting-based missing tag identification (TSMTI) method is developed to effectively identify missing tags and deactivate the remaining unknown ones. With tree-splitting, the identification time is greatly reduced and the reliability is further improved.

### 4.1. Phase I: Early-breaking-estimation-Based Unknown Tag Deactivation

**Figure 3:**
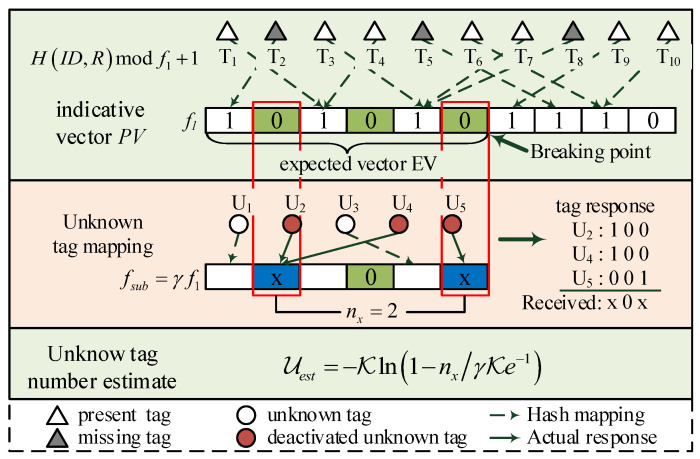
Early-breaking-estimation-based unknown tag deactivation.

In this phase, the reader executes a new EBUD algorithm to estimate the number of unknown tags in the first frame and deactivate them in subsequent frames. In the *i*-th frame of this phase, the reader first assigns known tags with hash mapping to construct an indicative vector PV. In detail, it generates the random seed *R*, sets frame size $f_{i}=K$ and calculates slot index for tag $T_{j}$ by

(2) $$\begin{array}{c} s\;=\;H\left(ID_{j},\;R\right)\;mod\;f_{i}\;+\;1, \end{array}$$

where H() is a hash function. Then, it generates PV with $f_{i}$ bits zeros and sets the *s*-th bit to be “1”, representing that the *s*-th slot is an *expected non-empty slot*. If there is no tag assigned, the reader sets the corresponding bit to be “0”, denoting an *expected empty slot*. As is shown on top of Figure 3, the constructed PV= “1 0 1 0 1 0 1 1 1 0”, i.e., only the 2nd, 4th, 6th and 10th slots are expected empty slots.

To effectively estimate the number of unknown tags, a new early-breaking-estimation method is introduced. The reader first sets the breaking point to obtain the expected vector EV. As is illustrated in Figure 3, the reader sets the breaking point to break PV into two parts, and the first $f_{sub}$ bits are expressed as the expected vector EV. Note that $f_{sub}=\left\lceil \gamma f_{1}\right\rceil$, where γ is the early-breaking factor ranging in [0,1] and ⌈·⌉ is the ceiling function. Then, it broadcasts the $Querye(R,\;f_{1},\;EV)$ command to inform tags with the random seed, frame size, and the expected vector. It should be noted that transmitting only the subvector of PV reduces time cost.

After receiving the *Querye* command, a tag checks the corresponding bit in EV and determine how to response. Each tag calculates the slot index *s* with (2) and checks the corresponding bit in EV. If EV(s) is “1” or *s* is greater than the length of $f_{sub}$, it will keep silent in the current frame. If EV(s) is “0”, the tag confirms that it is an unknown tag and constructs its response string with a bit-tracking response method. We denote the number of “0”s in EV by ${\bar{n}}_{0}$ and the number of “0” s prior to the *s*-th position by $n_{0}$. The tag first generates a ${\bar{n}}_{0}$ bit response string $R_{str}$ by setting the $\left(n_{0}+1\right)$-th bit to “1” and other bits be “0”s. Then, it replies $R_{str}$ to the reader and deactivates itself immediately. Since all known tags will be assigned to the “1” bit positions, and only unknown tags might map to the “0” bit positions, the reader can estimate the number of unknown tags by checking the response information of the expected empty slots in the next step. More specifically, as is shown in the middle of Figure 3, tags $U_{1}$ and $U_{3}$ are assigned into “1” bit positions of EV that they will keep silent in the current frame. Since tags $U_{2}$, $U_{4}$ and $U_{5}$ are assigned into the “0” bit positions, they will reply and deactivate themselves in this frame. Taking $U_{5}$ as an example, it constructs the response string as “001”, since there are 3 “0”s in EV and tag $U_{5}$ is assigned in the 3-rd “0” bit position. Similarly, the response strings of tags $U_{2}$ and $U_{4}$ are the same, i.e., “100”. With bit-tracking technology, the received message at the reader side in this frame is “x0x”, where “x” refers to a colliding bit. Notice that if there is only one tag response, the received message will have one “1” bit which is also regarded as an “x”.

By calculating the number of “x”s $n_{x}$, the reader estimates the number of unknown tags. Since tags are randomly assigned into slots, the probability that a tag is assigned to a specific slot is $1/f_{1}$. If the reader detects an “x” in the received message, it knows that at least one unknown tag replies in the *expected empty slot*. Recalling the construction of PV, the probability that no known tags are assigned to a specific bit position in PV is expressed as

(3) $$\begin{array}{c} p_{0}={\left(1-\frac{1}{f_{1}}\right)}^{K}\approx e^{-\frac{K}{f_{1}}}\approx e^{-1}. \end{array}$$

Similarly, the probability that at least one unknown tag is assigned in a specific position is calculated as

(4) $$\begin{array}{c} p_{u}=\left[1-{\left(1-\frac{1}{f_{1}}\right)}^{U}\right]\approx 1-e^{-\frac{U}{f_{1}}}=1-e^{-\frac{U}{K}}. \end{array}$$

Then, the probability that reader detects an “x” is given by

(5) $$\begin{array}{ccc} p_{x} & = & p_{0}\cdot p_{u}\approx e^{-1}\left(1-e^{-\frac{U}{f_{1}}}\right)=e^{-1}\left(1-e^{-\frac{U}{K}}\right). \end{array}$$

The expectation of the number of “x”s in the received message is calculated by $E\left(n_{x}\right)=\gamma f_{1}\cdot p_{x}$. Suppose the actual number of “x”s $n_{x}$ is approximately $E(n_{x})$, $n_{x}\approx E\left(n_{x}\right)=\gamma f_{1}\cdot p_{x}$. Substituting it to (5), the estimated number of unknown tags is calculated

(6) $$\begin{array}{c} U_{est}=-K\ln\left(1-\frac{n_{x}}{\gamma K\cdot e^{-1}}\right). \end{array}$$

Finally, the reader obtains the estimated number of unknown tags by collecting the responses from unknown tags.

In subsequent frames, the reader does similar operations to deactivate unknown tags. It assigns known tags into slots to construct the indicative vector PV and broadcasts $Queryd(R,\;f_{i},\;PV)$ command to tags. On receiving this command, tags that are assigned into the “0” bit positions in PV carry out hash mapping operations and deactivate themselves immediately. The deactivated unknown tags will not participate in Phase II. In general, the main discrepancies between the estimation and deactivation processes are in two folds. Firstly, in the Querye command, an expected vector EV copied from the first $\gamma f_{1}$ bits from PV is transmitted, whereas in Queryd, the full PV string is transmitted. Secondly, after receiving the Querye command, tags that are assigned into “0” bit positions in EV will reply to the reader. However, after receiving Queryd commands, tags will not reply to the reader, i.e., there are only the reader’s commands transmitted in each frame in the deactivation process. Tags that are assigned to the expected empty slots will deactivate themselves and keep silent.

### 4.2. Phase II: Tree-splitting-Based Missing Tag Identification

**Figure 4:**
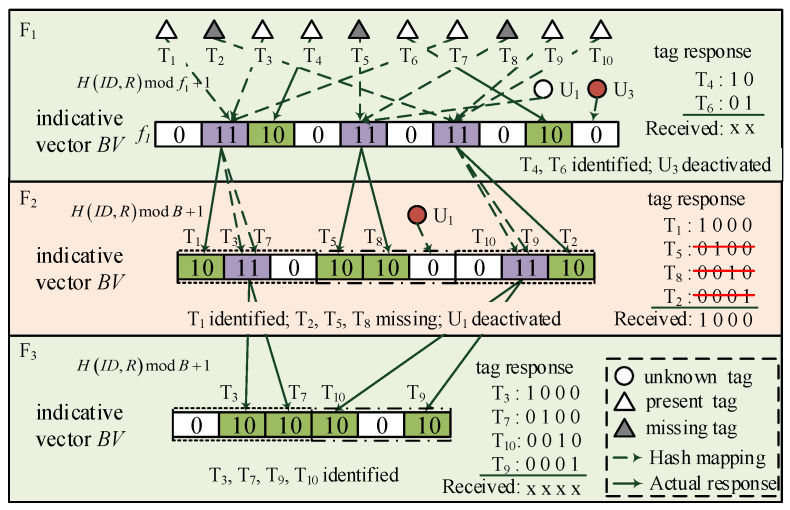
*B*-ary tree-splitting-based missing tag identification.

In this phase, the reader executes the *B*-ary tree-splitting method to quickly identify missing tags and deactivate the remaining unknown tags. Furthermore, the first frame is also different from the subsequent frames. In the first frame, the reader generates a random hash seed, sets frame length, and assigns known tags with hash mapping to construct the indicative vector BV as in Figure 4. Different from Phase I, three states should be indicated in BV: (i) If there is no tag assigned in a specific segment, this is an *expected empty slot* and denoted by a single “0” bit. (ii) If only one tag is assigned, this is an *expected singleton slot* and represented by “10”. (iii) Otherwise, this is an *expected collision slot* and denoted by “11”. For example, in $F_{1}$ of Figure 4, the 3-rdand9-th slots are two expected singleton slots, the 2-nd,5-th,and7-th slots are three expected collision slots, and others are expected empty slots. Then, the constructed indicative vector BV= “0 11 10 0 11 0 11 0 10 0”.

To facilitate the tree-splitting process, the reader keeps a counter for each known tag, i.e., $Ac(T_{j})$ for tag $T_{j}$. It will update the counter values after BV is constructed. If tag $T_{j}$ is assigned into an *expected singleton slot*, the reader sets $Ac(T_{j})=0$; if it is assigned into an *expected collision slot*, the reader calculates the number of “11” segments prior to the assigned position (denoted by $X_{11}$), and sets $Ac\left(T_{j}\right)=X_{11}+1$. Then, the reader broadcasts $Querym(R,\;f_{1},\;BV)$ and waits for tag responses.

After receiving this command, tag $T_{j}$ does the same hash mapping operations as the reader and checks the corresponding segment in BV as follows:

- If the assigned segment is “10”, the tag sets $Ac(T_{j})=0$ and prepares an ${\bar{X}}_{10}$ bits response string $R_{str}$, where ${\bar{X}}_{10}$ is the number of “10”s in BV. For instance, in frame $F_{1}$ of Figure 4, $Ac\left(T_{4}\right)=Ac\left(T_{6}\right)=0$, and ${\bar{X}}_{10}=2$;
- If the assigned segment is “11”, the tag does similar operations as the reader to obtain $X_{11}$, and sets $Ac\left(T_{j}\right)=X_{11}+1$. As is shown in frame $F_{1}$ of Figure 4, $Ac\left(T_{1}\right)=Ac\left(T_{3}\right)=Ac\left(T_{7}\right)=1$, $Ac\left(T_{5}\right)=Ac\left(T_{8}\right)=Ac\left(U_{1}\right)=2$ and $Ac\left(T_{2}\right)=Ac\left(T_{9}\right)=Ac\left(T_{10}\right)=3$;
- If the assigned segment is “0”, the tag determines that it is an unknown tag and will be deactivated. In frame $F_{1}$ of Figure 4, we can observe that $U_{3}$ is deactivated.

For a tag with $Ac(T_{j})=0$, it sets all bits of $R_{str}$ to be zero. It then counts the number of “10”s prior to its assigned segment in BV, and sets the corresponding bit in $R_{str}$ to be bit “1”. For example, in $F_{1}$ of Figure 4, tag $T_{4}$ is assigned into the first “10” segment in BV. It sets $R_{str}=\;$“10”. Similarly, tag $T_{6}$ is assigned into the second “10” segment in PV, so that it sets $R_{str}=$“01”. Then, the two tags reply $R_{str}$ and keep silent. After receiving tag responses, the reader decodes the received message as “xx” and confirms that tags $T_{4}$ and $T_{6}$ are present tags.

In subsequent frames, the reader identifies missing tags with a *B*-ary tree. In detail, the reader divides the *i*-th frame (i≥ 2) into multiple groups based on the number of expected collision slots in the (*i*-1)-th frame. Each group consists of *B* slots. The group index of each tag is determined by its counter value Ac. In each group, the reader assigns tags with s=H(ID,R)modB+1, and constructs indicative vector BV by concatenating the slot states in all groups. It then updates the counter values of all tags based on the constructed BV. For example, in $F_{2}$ of Figure 4, with *B* = 3, the reader assigns $T_{1}$, $T_{3}$ and $T_{7}$ in the first three slots because their Ac = 1; $T_{5}$ and $T_{8}$ with their Ac = 2 are assigned into the second group; $T_{2}$, $T_{9}$ and $T_{10}$ are assigned into the third group. The constructed indicative vector BV = “10 11 0 10 10 0 0 11 10”. Since $T_{3}$ and $T_{7}$ are assigned in the first expected collision slot; $T_{9}$ and $T_{10}$ are assigned in the second expected collision slot; other tags are assigned in the expected singleton slots, tags update their counter values as $Ac\left(T_{1}\right)=Ac\left(T_{5}\right)=Ac\left(T_{8}\right)=Ac\left(T_{2}\right)=0$, $Ac\left(T_{3}\right)=Ac\left(T_{7}\right)=1$, $Ac\left(T_{9}\right)=Ac\left(T_{10}\right)=2$. Next, the reader broadcasts Querym(R,B,BV) to tags.

On receiving reader’s Querym command, tag $T_{j}$ does similar hash operations as the reader and checks corresponding segments of the $Ac(T_{j})$-th group in BV. Then, it operates similarly to the tags in the first frame. If the tag is assigned into an expected singleton slot, it first checks the number of “10”s in BV, denoting by ${\bar{X}}_{10}$ and generates a response string $R_{str}$ with ${\bar{X}}_{10}$ zero bits. It then checks the number of “10”s prior to its assigned position and sets the corresponding bits in $R_{str}$ to “1” and replies to the reader. If the tag is assigned to an expected collision slot. It calculates the number of “11”s prior to its assigned position and updates Ac accordingly. If the tag is assigned to an expected empty slot, it deactivates itself.

For example, in $F_{2}$ of Figure 4, tags $T_{1},\;T_{3},\;\mathrm{and}\;T_{7}$ carry out hash mapping operations and check the first three segments, i.e., the first group, in BV. Tag $T_{1}$ is assigned to an expected singleton slot and tags $T_{3}$ and $T_{7}$ are assigned into an expected collision slot. Tag $T_{1}$ check the number of “10”s, generates $R_{str}=“1000”$ and replies to the reader. Tags $T_{3}$ and $T_{7}$ check the number of “11”s prior to their assigned position and update their counter values as $Ac\left(T_{3}\right)=Ac\left(T_{7}\right)=1$. In the 4-th to 6-th segments, tag $U_{1}$ is assigned into an expected empty slot that will be deactivated. In the 7-th to 9-th segments, tags $T_{9}$ and $T_{10}$ are assigned into the same expected collision slot. They update $Ac\left(T_{9}\right)=Ac\left(T_{10}\right)=2$. Since tags $T_{2}$, $T_{5}$ and $T_{8}$ are missing, only tag $T_{1}$ will reply in this frame.

After receiving tags’ responses, the reader determines that tag $T_{1}$ is present, and tags $T_{2}$, $T_{5}$, and $T_{8}$ are missing. Similarly, the reader confirms that tags $T_{3}$, $T_{7}$, $T_{9}$ and $T_{10}$ are present in $F_{3}$. If there are no collision slots in $F_{3}$, it means all tags are identified. Then, the reader terminates the current reading round. Otherwise, it splits collision slots and repeats the identification process in subsequent frames. With tree-splitting, colliding tags are more easily separated and the identification process is effectively accelerated.

## 5. Performance Analysis

**Figure 5:**
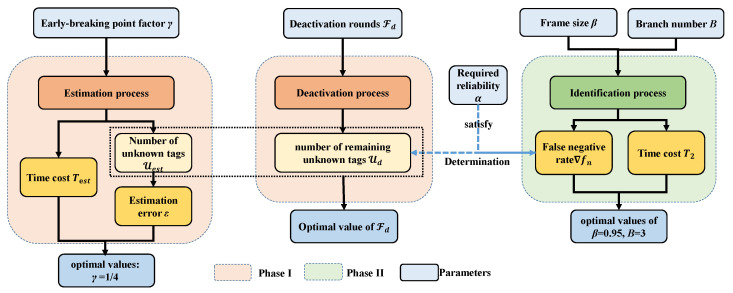
The logic diagram of performance analysis: firstly, time cost of Phase I is analyzed and the early-breaking factor γ is determined through balancing estimation error ϵ and time cost $T_{est}$; secondly, time cost $T_{2}$ and false negative rate $\nabla_{fn}$ of Phase II is analyzed and the optimal frame parameter β and branch number *B* are obtained; finally, the number of frames in Phase I $F_{d}$ is determined to deactivate enough unknown tags based on the estimated unknown tag number $U_{est}$ and required reliability α.

In this section, we first analyze the deactivation phase and optimize the early-breaking factor γ to balance the time cost and estimation error of EBUD. Next, we analyze the identification phase and optimize the frame parameter β and the branch number *B*. Then, the false negative rate of the identification phase is analyzed, since the false negative rate is affected by the number of unknown tags participating in Phase II, the number of frames needed in Phase I is determined by making use of the estimated unknown tag number and the required reliability to deactivate enough unknown tags. More specifically, Figure 5 illustrates the main logic of our analysis.

### 5.1. Time Cost of Phase I

In Phase I, a new EBUD method is developed to effectively estimate the number of unknown tags and deactivate them. Time cost of EBUD is given by

(7) $$\begin{array}{c} T_{1}=T_{est}+T_{dea}, \end{array}$$

where $T_{est}$ and $T_{dea}$ are the time costs of the estimation and deactivation processes, respectively.

In the estimation process, each frame consists of the transmission of the reader’s Querye() command and unknown tags’ responses. As given in Section 4.1, $Querye(\;R,\;f_{1},\;EV)$ command consists of a 4-bit command type string, a 16-bit hash seed, a 16-bit frame size, and a $\gamma f_{1}$-bit expected vector. On the tag side, unknown tags are assigned to expected empty slots that will reply immediately. With the bit-tracking response, the length of a response message is the number of expected empty slots indicated in EV. Since the probability of a specific slot to be empty is ${\left(1-\frac{1}{f_{1}}\right)}^{K}$, the number of expected empty slots is $\gamma f_{1}{\left(1-\frac{1}{f_{1}}\right)}^{K}$. Thus, the time cost is given by

(8) $$\begin{array}{ccc} T_{est}= & \{\;\underset{reader\;request}{\underset{︸}{\left\lceil \frac{\gamma f_{1}+16\cdot 3+4}{96}\right\rceil }} & +\underset{tag\;responses}{\underset{︸}{\left\lceil \frac{\gamma f_{1}{\left(1-\frac{1}{f_{1}}\right)}^{K}}{96}\right\rceil }}\;\}t_{id}, \end{array}$$

where $f_{1}=K$ and $t_{id}$ is time cost for transmitting a 96-bit string. It should be noted that both the reader’s request command and tags’ responses are divided into 96-bit segments to facilitate transmission.

In the estimation process, two indexes, $T_{est}$ and estimation error ϵ, are adopted to determine the early-breaking factor γ. Define the estimation error as

(9) $$\begin{array}{c} \epsilon =abs\left(\frac{U_{est}-U}{U}\right), \end{array}$$

where abs(·) returns the absolute value of a number. Table 1 gives the statistic results averaged from 100 tests to demonstrate how γ affects these two indexes. As is shown, with smaller γ, the estimation error increases and the time cost decreases. To balance the two indexes and provide reasonable estimation accuracy, we set γ=1/4 in EBUD.

In the deactivation process, each frame only consists of the transmission of the reader’s request command $Queryd(R_{i},\;f_{i},\;PV)$. The time cost is calculated by

(10) $$\begin{array}{c} T_{dea}=\sum_{i=1}^{F_{d}}\left\lceil \frac{f_{i}+16\times 3+4}{96}\right\rceil t_{id} \end{array}$$

Substituting (8) and (10) into (7), the time cost of Phase I is obtained.

### 5.2. Time Cost of Phase II

In Phase II, a new *B*-ary tree-splitting-based missing tag identification (TSMTI) method is developed to quickly identify missing tags. The time cost of Phase II consists of two parts, i.e.,

(11) $$\begin{array}{c} T_{2}=T_{r}+T_{t}, \end{array}$$

where $T_{r}$ and $T_{t}$ are the time costs of transmitting reader requests and tag responses in Phase II, respectively.

In frame $F_{1}$, a tag is randomly assigned into an expected slot indicated in BV, and the probability is given by $1/f_{1}=1/\left(\beta K\right)$. In subsequent frames, tags assigned in the same expected collision slot are split into *B* subgroups. In Figure 4, the splitting process can be viewed as a single search applied to a tree whose root node has $f_{1}$ children, and all subsequent nodes have *B* children. Inspired by [35], we consider these root nodes for the individual tree searches to be at level 0, and the *i*-th level of the tree can be viewed as the (i+1)-th frame in Phase II. In the *i*-th level, the search probes over subintervals of size $B^{i}$. Thus, a tag is assigned to a specific slot of the *i*-th level given by

(12) $$\begin{array}{c} p=\left(\frac{1}{f_{1}}\right)B^{-i}=\frac{1}{\beta KB^{i}}. \end{array}$$

Then, the probability that *j* out of K tags fall into a particular slot of level *i* is

(13) P(j,K,i)=Kjpj(1−p)K−j.

Probabilities that a slot is an expected empty, a singleton or a collision slot are separately given as follows:

(14) $$\begin{array}{ccc} P_{empt} & = & P\left(0,K,i\right)={\left(1-p\right)}^{K}, \end{array}$$

(15) $$\begin{array}{ccc} P_{sing} & = & P\left(1,K,i\right)=Kp{\left(1-p\right)}^{K-1}, \end{array}$$

(16) $$\begin{array}{ccc} P_{coll} & = & P\left(j>1,K,i\right)=1-{\left(1-p\right)}^{K}-Kp{\left(1-p\right)}^{K-1}. \end{array}$$

Let $q_{i}$ be the probability that a particular slot at level *i* is visited in the splitting process. In level *i*, a slot is visited only when its parent experiences a collision. Otherwise, if its parent slot is empty or a singleton, it cannot generate subgroups. Then, we have

(17) $$\begin{array}{c} q_{i}=\left\{\begin{array}{cc} P\left(j>1,K,i-1\right) & i\geq 1\\ 1 & i=0 \end{array}\right.. \end{array}$$

It can be noted that all slots at level 0 will be probed; hence, $q_{0}=1$. In the *i*-th level, the average number of expected slots to be visited is determined by summing $q_{i}$ over all sub-intervals which is equal to $\beta KB^{i}$, i.e.,

(18) $$\begin{array}{c} S_{i}\left(K\right)=\beta KB^{i}q_{i}. \end{array}$$

The reader broadcasts $Querym(\;R,\;f_{1},\;BV)$ in the first frame or Querym(R,B,BV) in subsequent frames to tags. When a tag is assigned to an expected singleton slot, it will reply to the reader. For each frame, number of segments in BV is obtained by (18). Since each level refers to one frame and the state of each slot is indicated by at most 2 bits in BV, the time cost for transmitting the reader’s request commands can be approximated by

(19) $$\begin{array}{c} T_{r}=\sum_{i=1}^{F_{m}}T_{r\_f_{i}}=\sum_{i=0}^{F_{m}-1}\left\lceil \frac{2S_{i}\left(K\right)+52}{96}\right\rceil t_{id}=\sum_{i=0}^{F_{m}-1}\left\lceil \frac{2\beta KB^{i}q_{i}+52}{96}\right\rceil t_{id}, \end{array}$$

where $T_{r\_f_{i}}$ is time cost for transmitting reader command in the *i*-th frame, and $F_{m}$ is the number of frames needed in Phase II.

If a tag is resolved in a level higher than *i* in the tree, then it will also be resolved in level *i* [35]. Hence, by counting all singleton slots in level *i*, we are accounting for all singleton slots visited up to and including those at level *i*. The number of identified tags in level *i* is equal to the number of singleton slots in *i* level minus the number of singleton slots in level i−1. Then, the number of identified tags in level *i* is calculated as:

(20) $$\begin{array}{c} K_{i}^{*}=\left\{\begin{array}{cc} \beta KB^{i}\left[P\left(1,K,i\right)-P\left(1,K,i-1\right)\right] & i\geq 1\\ \beta KB^{i}P\left(1,K,0\right) & i=0 \end{array}\right.. \end{array}$$

As is shown in Figure 4, with bit-tracking technology, the length of tags’ response message in each frame equals the number of expected singleton slots indicated in BV. In the *i*-th frame of Phase II, the time cost for transmitting tag responses is given by

(21) $$\begin{array}{c} T_{t\_f_{i}}=\left\lceil \frac{K_{i-1}^{*}}{96}\right\rceil t_{id}. \end{array}$$

With (20) and (21), we have

(22) $$\begin{array}{c} T_{t}=\sum_{i=1}^{F_{m}}T_{t\_f_{i}}=\sum_{i=0}^{F_{m}-1}\left\lceil \frac{K_{i}^{*}}{96}\right\rceil t_{id}=\beta K\left\{P\left(1,K,0\right)\;+\;\sum_{i=1}^{F_{m}-1}B^{i}\left[P\left(1,K,i\right)\;-\;P\left(1,K,i-1\right)\right]\right\} \end{array}$$

Because Phase II terminates when all known tags are identified, $F_{m}$ should meet the requirement that

(23) $$\begin{array}{c} \left\lceil \sum_{i=0}^{F_{m}-1}K_{i}^{*}\right\rceil =K\Rightarrow \;F_{m}. \end{array}$$

**Figure 6:**
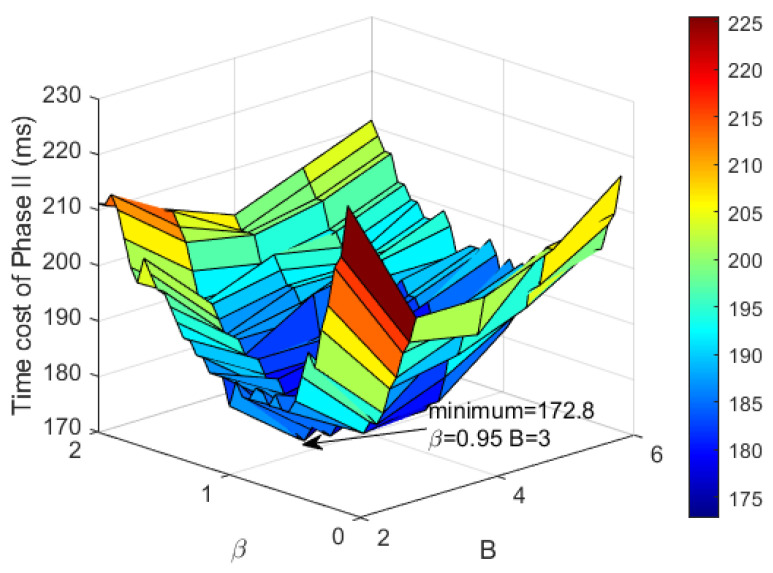
Time cost of Phase II when β and *B* changes.

Substituting (19), (22) and (23) to (11), time cost of TSMTI is obtained. In Phase II, two parameters affect the performance of TSMTI, i.e., frame factor β, and branch number *B*. Figure 6 gives the numerical results of $T_{2}$ when β and *B* changes. As can be observed, $T_{2}$ decreases when β ranges from 0.1 to 0.95, and increases when β>0.95. In the meantime, when B=3, $T_{2}$ is smaller than other settings of *B*. Therefore, the near-optimal parameters are given by β=0.95 and B=3.

### 5.3. False Negative Rate

In Phase II, if a missing tag is assigned to the expected singleton slot and at least one unknown tags happen to be assigned to the same slot, the missing tag will be falsely identified as present. With (15), the number of misidentified missing tags at the *i*-th level of Phase II is given by

(24) $$\begin{array}{c} M_{fls,i}=\underset{missing\;tag}{\underset{︸}{M_{i}^{*}}}\underset{unknown\;tag}{\underset{︸}{\left[1-{\left(1-p\right)}^{U_{i}}\right]}}. \end{array}$$

Here, $M_{i}^{*}$ and $U_{i}$ are the numbers of missing tags to be identified and unknown tags participating in the *i*-th level of Phase II. In (24), the first segment represents the number of expected singleton slots with missing tags which equals to $M_{i}^{*}$, and the second segment refers to the probability that at least one unknown tag selects this slot. Suppose that the missing tags are evenly distributed, the probability that one known tag is missing is $\frac{M}{K}$. Based on (20), the number of missing tags to be identified in level *i* is expressed as:

(25) $$\begin{array}{c} M_{i}^{*}=\frac{M}{K}\cdot K_{i}^{*}. \end{array}$$

Only when an unknown tag is assigned to an expected collision slot in level i−1 will the tag participate in level *i*. Based on (16), the number of remaining unknown tags in level *i* is given by

(26) $$\begin{array}{c} U_{i}=U_{i-1}P\left(j>1,K,i-1\right)\;\leq U_{0}\left[1-{\left(1-p\right)}^{K}\;-\;Kp{\left(1-p\right)}^{K-1}\right]. \end{array}$$

Here, $U_{0}$ is the number of unknown tags participating in level 0, i.e., the first frame of Phase II. It equals the number of remaining unknown tags $U_{d}$ after Phase I, i.e., $U_{0}=U_{d}$. Substituting (25) and (26) into (24), number of misidentified missing tags at level *i* is obtained. Finally, the false negative rate of TSMTI is given by

(27) $$\begin{array}{c} \nabla_{fn}=\frac{M_{fls,i}}{M}=\frac{\sum_{i=0}^{F_{m}-1}M_{fls,i}}{M}\leq \sum_{i=0}^{F_{m}-1}\frac{K_{i}^{*}}{K}\left\{1-{\left(1-p\right)}^{U_{d}\left[1-{\left(1-p\right)}^{K}\;-\;Kp{\left(1-p\right)}^{K-1}\right]}\right\}. \end{array}$$

The false negative rate of TSMTI is affected by $U_{d}$. To analyze the effect, we set the remaining unknown tag ratio $r_{ud}=\frac{U_{d}}{K}$, i.e., the percentage of remaining unknown tags to the known ones. Based on our analysis, Table 2 illustrates the numerical values of $\nabla_{fn}$ when $r_{ud}$ varies.

With (1), we have $\nabla_{fn}<1-\alpha$. When the required reliability α=0.9, $\nabla_{fn}<0.1$. According to Table 2, the allowed remaining unknown tag ratio $r_{ud}\leq 0.15$ and we set $r_{ud}=0.10$ to meet the requirement. Similarly, when α=0.95 (resp. 0.99), we set $r_{ud}$ as smaller than 0.05 (resp. 0.01), respectively. Therefore, the number of remaining tags should meet

(28) $$\begin{array}{c} U_{d}\leq \left\{\begin{array}{c} 0.1K,\;\;\;\alpha \leq 0.90\\ 0.05K,\;\;0.90<\alpha \leq 0.95\\ 0.01K,\;\;0.95<\alpha \leq 0.99 \end{array}\right. \end{array}$$

### 5.4. Determination of $F_{d}$ in Phase I

With the required number of remaining unknown tags after Phase I, the number of frames needed to deactivate enough unknown tags can be calculated. Recalling the deactivation process of Phase I, when an unknown tag is assigned to the expected empty slot indicated in PV, it will deactivate itself. Thus, in the i-th frame of the deactivation process, the number of newly deactivated unknown tags $U_{i}^{*}$ is given by

(29) Ui*=Uifi11fi1−1fiK≈Uie−K/fi=Uie−1,

where $U_{i}$ is the number of unknown tags participating in the i-th frame and the frame size $f_{i}=K$. The initial value $U_{1}$ = U. With recursive resolving, the number of remaining unknown tags $U_{d}$ after $F_{d}$ frames can be calculated as follows,

(30) $$\begin{array}{ccc} U_{d} & = & U_{F_{d}}-U_{F_{d}}^{*}=U_{F_{d}}\left(1-\;e^{-1}\right)=U_{F_{d}-1}{\left(1-\;e^{1}\right)}^{2}\\ & = & U_{1}{\left(1-e^{-1}\right)}^{F_{d}}=U{\left(1-e^{-1}\right)}^{F_{d}}. \end{array}$$

With the estimated unknown tag number, $F_{d}$ is obatined by,

(31) $$\begin{array}{c} F_{d}\approx \frac{\ln\left(U_{d}/U_{est}\right)}{\ln\left(1-e^{-1}\right)}. \end{array}$$

Substituting (28) into (31), the number of frames needed in the deactivation process of Phase I is obtained, i.e.,

(32) $$\begin{array}{c} F_{d}\geq \left\{\begin{array}{c} \frac{\ln\left(\frac{0.1K}{U_{est}}\right)}{\ln(1-e^{-1})},\;\;\alpha \leq 0.9\\ \frac{\ln\left(\frac{0.05K}{U_{est}}\right)}{\ln(1-e^{-1})},\;0.9<\alpha \leq 0.95\\ \frac{\ln\left(\frac{0.01K}{U_{est}}\right)}{\ln(1-e^{-1})},\;0.95<\alpha \leq 0.99 \end{array}\right. \end{array}$$

In conclusion, as is shown in Figure 5, to determine the number of deactivation frames $F_{d}$ in Phase I, the reader first executes the estimation process to estimate the number of unknown tags $U_{est}$ with (6). It then calculates $F_{d}$ with (32) based on the estimated unknown tag number and the reliability requirement.

## 6. Evaluation

In this section, we first describe the simulation configurations and then evaluate the performance of our proposed ETMTI protocol in different phases: (1) Phase I, the deactivation phase; (2) Phase II, the identification phase, and (3) the overall process, separately. The time cost is the essential metric to show system effectiveness, and the false negative rate is the important metric for system reliability. As shown in Table 3, we will simulate different scenarios for different phases: Phase I in $S_{11}$, $S_{12}$, $S_{13}$ and $S_{14}$ and Phase II in $S_{21}$ and $S_{22}$. Next, the performance of time cost and false negative rate of the overall identification process under scenarios $S_{31}$, $S_{32}$ and $S_{33}$ are given. Meanwhile, the results of some best-performing benchmarks are presented for a comprehensive comparison.

### 6.1. Simulation Configurations

In the simulation, a typical RFID system that consists of a reader, K known tags, and U unknown ones are considered. In a known tag set, M tags are missing. To simulate different scenarios in large-scale RFID systems, these parameters including K, M, and U can be defined by users. There are also other inputs: a required reliability α, unknown ratio $r_{u}=U/K$ and missing ratio $r_{m}=M/K$. The reader can retrieve known tags’ information from the backend database but has no prior knowledge about the unknown ones. For a fair comparison, we set the configuration parameters similar to previous works [6,7,11], each tag has a unique ID with 96-bit length, and the data rate between reader and tags is 40 Kbps, since the index values of our tags are mapped through our specially designed hash function, which can maintain an even distribution of the output index values even if the tag IDs are not evenly distributed. Hence, we can suppose the tag ID are default uniform distribution. The transmitted message between the reader and tags is divided into 96-bit segments and each segment takes $t_{id}=2.4$ ms. This configuration also adheres to Philip’s I-Code [36], enabling us to simulate the ETMTI protocol’s performance under real-world conditions. As in the literature [12,13], communications between reader and tags are assumed to be error-free first, and the effect of unreliable channel on the identification has been analyzed in Section 6.2.

In the simulation, the performance of our proposed ETMTI protocol is compared with the most related ERMI [13], BMTD [6], CBMTD [7], CRMTI [12], PCMTI [11], and SFMTI [10] protocols. ERMI is the most representative missing tag identification protocol that considers the presence of both known and unknown tags. BMTD and CBMTD present the most related unknown tag deactivation methods. PCMTI is the first related work that uses bit-tracking technology in missing tag identification. SFMTI is proposed to reconcile some expected collision slots into singleton slots and filter out the expected empty slots as well as the unreconcilable collision slots. CRMTI is the most efficient missing tag identification protocol for situations with only known tags. The simulation is conducted with Matlab R2019b, and each result is averaged over 100 tests.

### 6.2. Time Cost of Phase I

In this phase, the reader estimates the number of unknown tags and deactivates them with multiple frames. To deactivate enough unknown tags, the number of frames of this phase is determined with (32). In the simulation, we evaluate the time cost of Phase I in four scenarios as described in Table 3. $S_{11}$ and $S_{12}$ represent the situation where the number of known tags K within the communication range increases, that is, the density of known tags increases. Situations $S_{13}$ and $S_{14}$ represent simulations when the density of unknown tags $r_{u}$ increases. Additionally, the required reliabilities of $S_{11}$ and $S_{14}$ are 0.99, higher than $S_{12}$ and $S_{13}$, meaning lower required false negative rate. Generally speaking, the scenarios are set to test the stability and scalability of the unknown tag deactivation protocols.

**Figure 7:**
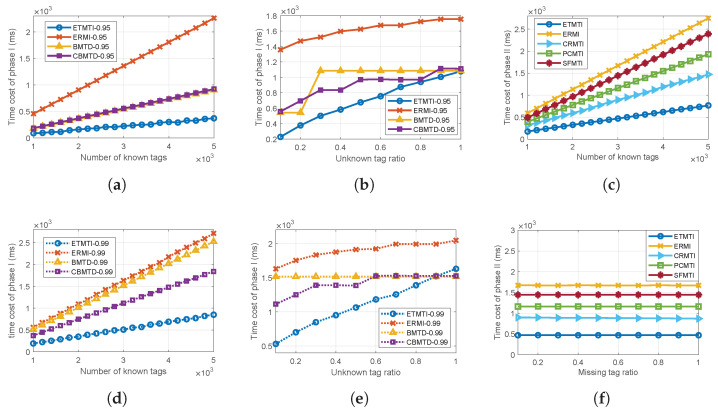
Time cost: (**a**) time cost of Phase I in scenario $S_{11}$; (**d**) time cost of Phase I in scenario $S_{12}$; (**b**) time cost of Phase I in scenario $S_{13}$; (**e**) time cost of Phase I in scenario $S_{14}$; (**c**) time cost of Phase II in scenario $S_{21}$; (**f**) time cost of Phase II in scenario $S_{22}$.

Since missing tags do not affect the deactivation process, the missing tag ratio $r_{m}$, i.e., the fraction of a number of missing tags to that of known tags, is set to be 0. The time cost of ETMTI in Phase I is compared with the most related ERMI, BMTD, and CBMTD protocols, and the comparative results are presented in Figure 7a,b,d,e. It should be noted that the unknown tag number estimation process in BMTD and CBMTD is neglected in the simulation since the specified unknown tag number estimation method in their works is complicated and time-consuming.

*(1) Impact of Number of Known Tags:* As shown in Figure 7a,d, the time cost of Phase I increases with the number of known tags. With a fixed unknown tag ratio, the number of unknown tags increases with that of known ones. To meet the required reliability, longer frame lengths and more frames are needed to deactivate enough unknown tags in the deactivation process. Comparing the simulation results in Figure 7a with those in Figure 7d, we can observe that with higher reliability requirements, the time cost of Phase I also increases. Among the comparative protocols, ETMTI always takes the shortest time to deactivate enough unknown tags, and ERMI takes the longest time. Thanks to the early-breaking and bit-tracking response strategies in ETMTI, the time used for unknown tag number estimation is greatly reduced. Thus, it takes much less time than other protocols, whereas ERMI takes more time to estimate the number of unknown tags since it executes the whole estimation frame. Therefore, ERMI takes more time than ETMTI.

Taking advantage of multiple hash functions, BMTD uses bloom filters to deactivate unknown tags. In BMTD, the number of frames is determined by minimizing the overall identification time, and the performance of the deactivation phase is not optimized. As demonstrated in Figure 7a,d, BMTD takes a longer time than ETMTI, but a shorter time than ERMI. In order to reduce the number of hash functions used in BMTD, CBMTD proposed a compressed method to reduce the time cost of the deactivation process. However, this method may not always work well. In Figure 7d, one can observe that the time cost of BMTD is larger than CBMTD when α=0.99, whereas as is shown in Figure 7a, BMTD and CBMTD take almost the same time when α=0.95.

*(2) Impact of Unknown Tag Ratio:* Next, as demonstrated in Figure 7b,e that time cost of Phase I increases with an unknown tag ratio. One can observe that ETMTI takes the shortest time and ERMI takes the longest time thanks to the fewer messages needed to estimate the number of unknown tags resulting in less time cost. Moreover, the estimated tag number and number of frames of the deactivation process in ETMTI are appropriately set. In ERMI, the frame size of the estimation process is set to be the number of known tags. With a slot-by-slot reply method, more time is needed to estimate unknown tags. Therefore, ERMI takes longer time than ETMTI. In BMTD, a few frames are used in the deactivation process, but the frame length is set to be very long to deactivate more unknown tags in each frame. Thus, it takes more time than ETMTI, especially when the unknown tag ratio is small. With compressed filters, CBMTD takes a shorter time than BMTD in most cases.

In Phase I, our proposed EBUD method of ETMTI protocol has successfully estimated and deactivated unknown tags in multiple scenarios, including when the number of known tags or unknown tag ratio increases. The proposed EBUD shows better performance than other comparative protocols to deactivate unknown tags. These findings underscore the efficiency of our proposed early-breaking and bit-tracking strategies in handling unknown tags.

### 6.3. Time Cost of Phase II

In this phase, a missing tag identification protocol is executed to verify the presence of known tags and identify missing ones. We evaluate the time cost of Phase II in two scenarios as described in Table 3. $S_{21}$ and $S_{22}$ exhibit the TSMTI under the scenarios where the number of known tags K increases and missing tag ratio $r_{m}$ increases, respectively. They are set to test the effectiveness and scalability of the missing tag identification protocols. Since the unknown tags do not affect the time cost of Phase II, $r_{u}$ is set to be 0. The simulation results of ETMTI are compared with the most related ERMI and CRMTI protocols.

*(1) Impact of the number of known tags:* As is illustrated in Figure 7c, the time cost of the missing tag identification protocols increases with the number of known tags. Among the comparative protocols, ETMTI takes the least time to identify all tags, and ERMI takes the most time.

*(2) Impact of missing tag ratio:* As is shown in Figure 7f, time costs of the comparative protocols remain unchanged when the missing tag ratio changes. In this phase, the reader has to verify the presence of all known tags and that the identification time is only affected by the number of known tags. With a fixed K, the time cost of Phase II remains unchanged. In the two scenarios, we observe that ETMTI always takes the least time for missing tag identification of Phase II.

The main reasons are as follows. In ETMTI, a new *B*-ary tree-splitting method is proposed to split colliding tags into smaller groups in a layered structure. The collision probability reduces as the number of layers increases resulting in increased utilization of the indicative vector, whereas, ERMI, CRMTI, PCMTI, and SFMTI adopt the Aloha-based method to randomly assign tags repeatedly. In each frame, the collision probability is high. Although CRMTI and SFMTI use collision-resolving methods to increase the utilization of indicative vectors, it still takes longer time than ETMTI. Moreover, tag response strategies used in the comparative protocols are also different. In ERMI and SFMTI, the tag replies with a 1-bit short response in the expected singleton slot. With collision resolving and bit-tracking strategies, CRMTI allows multiple tags to reply with customized responses simultaneously in the expected resolvable collision slot. PCMTI verifies two tags in one short response slot simultaneously. Thus, the time cost for tag response in CRMTI is smaller than that in PCMTI, ERMI, and SFMTI. Extending the bit-tracking strategy to all slots, ETMTI further reduces the overhead of each slot and the time cost of ETMTI is smaller than other comparative protocols.

In Phase II, our proposed TSMTI method of ETMTI protocol has successfully identified known tags in multiple scenarios, including when the number of known tags or missing tag ratio increases. With the *B*-ary tree-splitting and bit-tracking strategies, the proposed TSMTI takes the least time than other comparative protocols. 

### 6.4. Effect of Unreliable Channels

In the above analysis, the channels between the reader and tags are assumed to be error-free. We further investigate the effect of the imperfect channels, where two main factors may cause misidentification, namely the detection probability $p_{d}$ and the bit error rate $p_{e}$. These factors may cause two types of misidentification in the proposed protocol, i.e., the false positive and false negative tags. A false positive tag occurs when a present tag is confirmed as a missing one. On the other hand, a false negative tag occurs if a missing tag is mistakenly detected as present.

**Figure 8:**
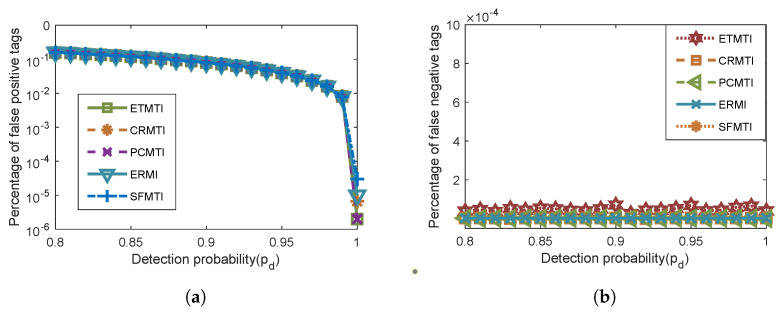
Effect of unreliable channels: (**a**) percentage of false positive tags vs. detection probability; (**b**) percentage of false negative tags vs. detection probability.

The performance under unreliable channels is evaluated, and the percentages of false positive and false negative tags are presented in Figure 8a,b, respectively. Note that bit errors in most indoor scenes where the distance between the reader and the tag is often less than 10 m rarely occur, and the bit error rate is less than $10^{-6}$ [37]. We adopt this value in our simulation. As shown in Figure 8a, the percentages of false positive tags of all the compared protocols decrease when the detection probability increases and the values are essentially identical. Moreover, Figure 8b depicts that the percentages of false negative tags of the compared protocols remain almost unchanged when $P_{d}$ changes. Although our proposed ETMTI expresses a slightly higher value, the order of magnitude of the false negative rate is less than $10^{-4}$. The percentage of false negative tags remains at a very low level, which has a negligible impact on the identification process. As is demonstrated, the detection probability and bit error rate have similar effects on the identification process of all the comparing protocols.

### 6.5. Performance of the Overall Process

**Figure 9:**
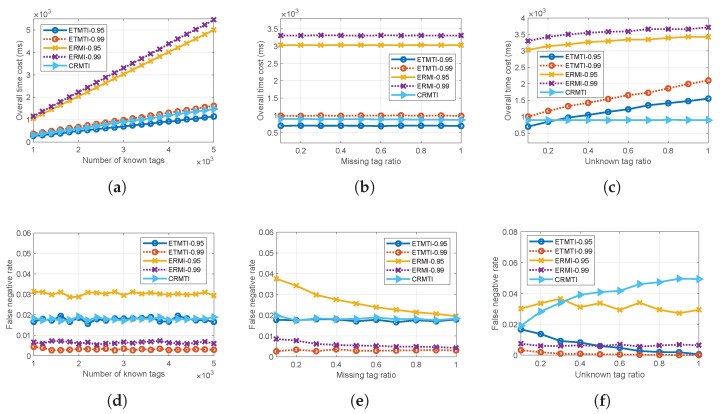
Performance of the overall process: (**a**) time cost vs. number of known tags in scenario $S_{31}$; (**b**) false negative rate vs. number of known tags in scenario $S_{31}$; (**c**) time cost vs. missing tag ratio in scenario $S_{32}$; (**d**) false negative rate vs. missing tag ratio in scenario $S_{32}$; (**e**) time cost vs. unknown tag ratio in scenario $S_{33}$; (**f**) false negative rate vs. unknown tag ratio in scenario $S_{33}$.

In this part, we evaluate the time cost and false negative rate of the overall process in three scenarios as described in Table 3. To simulate large-scale RFID deployments, the scenarios $S_{31}$, $S_{32}$ and $S_{33}$ represent the number of known tags K, missing tag ratio $r_{m}$ and unknown tag ratio $r_{u}$ increase, respectively. The performance of ETMTI is compared with the most related ERMI and CRMTI protocols and the comparative results are illustrated in Figure 9. For ETMTI and ERMI, simulation experiments when α=0.95 and α=0.99 are separately conducted in each scenario.

*(1) Impact of Number of Known Tags:* As is shown in Figure 9a, the overall time costs of all protocols increase with the number of known tags. Benefiting from the bit-tracking strategies, ETMTI and CRMTI take a much shorter time than ERMI. When α=0.95, ETMTI takes the least time than other comparative protocols. When α=0.99, ETMTI takes a little bit longer time than CRMTI. As can be observed in Figure 9a, time costs of ETMTI and ERMI increase with the required reliability. With a larger α, more time is needed in Phase I to deactivate enough unknown tags.

Figure 9d presents that false negative rates of all comparative protocols keep unchanged when the number of known tags varies. As is shown, false negative rates of ETMTI and ERMI decrease as the required reliability increases. When α=0.95, the false negative rate of ERMI is about 0.03, and that of ETMTI is reduced below 0.02. When α=0.99, the false negative rate of ERMI is about 0.007, and that of ETMTI is about 0.004. Both ETMTI and ERMI achieve the required reliability, and ETMTI always has a smaller false negative rate than ERMI in the same condition. We can also observe that the false negative rate of CRMTI is almost the same as that of ETMTI (when α=0.95). In this scenario, the unknown tag ratio is so small that the unknown tags have little effect on the identification process of CRMTI. Thus, the false negative rate is low. Since CRMTI does not deal with unknown tags, the lowest false negative rate it can achieve is around 0.19.

*(2) Impact of Missing Tag Ratio: *Figure 9b,e present the overall time cost and false negative rate separately when the missing tag ratio varies. As is demonstrated in Figure 9b, time costs of the comparative protocols remain unchanged when the missing tag ratio increases. In ERMI and ETMTI, the overall process is affected by the number of known and unknown tags, as well as the required reliability. With larger α, the time costs of ETMTI and ERMI increase since the reader needs more time to deactivate enough unknown tags. CRMTI is only affected by the number of known tags. Therefore, the overall time costs of the comparative protocols do not change with the missing tag ratio. In general, we can observe that ETMTI (when α=0.95) takes the shortest time, CRMTI takes longer time than ETMTI (when α=0.95), but a little bit lower time than ETMTI (when α=0.99), and ERMI always takes the longest time.

In Figure 9e, ETMTI (when α=0.99) has the least false negative rates, and ERMI (when α=0.95) has the worst performance. We can also observe that CRMTI shows similar performance with ETMTI (when α=0.95), but it takes more time as is shown in Figure 9b. When α=0.99, ERMI shows a slightly higher false negative rate than ETMTI, but the increased time cost is too much. It should be noted that the false negative rate of ERMI decreases as the missing tag ratio increases. In an expected singleton slot, if the assigned known tag is missing and one or more unknown tags are assigned to this slot. The missing tag will be falsely identified as present, resulting in a false negative event. In this scenario, the number of unknown tags is a fixed small value. As the missing tag number increases, the percentage of falsely identified missing tags decreases, so the false negative rate decreases accordingly.

*(3) Impact of Unknown Tag Ratio: *Figure 9c,f exhibit the overall time cost and false negative rate when the unknown tag ratio changes, respectively. As is shown in Figure 9c, the time cost of CRMTI remains unchanged since it is only affected by the number of known tags. However, in ETMTI and ERMI, as the unknown tag ratio increases, more time is needed to deactivate enough unknown tags in Phase I. Thus, the overall time costs of ETMTI and ERMI increase with an unknown tag ratio. Similarly, their time costs also increase with the required reliability. Moreover, as is demonstrated in Figure 9c, when the unknown tag ratio is small, ETMTI (when α=0.95) takes the least time. As the unknown tag ratio increases, ETMTI (when α=0.95) takes more time than CRMTI.

In Figure 9f, ETMTI (when α=0.99) has the least false negative rate than other comparative protocols. ERMI (when α=0.99) has a higher false negative rate than ETMTI (when α=0.99). CRMTI and ERMI (when α=0.95) show the worst performance. We can observe that the false negative of ETMTI decreases as the unknown tag ratio increases. With more unknown tags, ETMTI needs more frames to deactivate them in Phase I. This is in accordance with the increasing trends of overall time cost in Figure 9c. The increased number of frames further increases the percentage of deactivated unknown tags resulting in a reduced number of unknown tags that participate in Phase II. Therefore, the false negative rate of ETMTI decreases with the increase in the unknown tag ratio.

In ERMI, the false negative rate also decreases as the unknown tag ratio increases, but the decrease rate is very small. Since the number of deactivated unknown tags of ERMI is not as much as that in ETMTI, the decreased false negative rate is not obvious. Note that the fluctuations in ERMI are mainly caused by the inaccurate estimate of the unknown tag number. Without any deactivation strategy, the false negative rate of CRMTI increases with an unknown tag ratio. To sum up, ETMTI exhibits better performance in terms of time cost and false negative rate than the comparative benchmark works.

In summary, the simulation results reveal that our proposed ETMTI protocol provides a more efficient and reliable solution for missing tag identification. Through evaluating the performance of our protocol in different scenarios, our protocol’s performance remains efficient and reliable even as the number of known tags, unknown tags, or missing tags in the system increases, ensuring its suitability for large-scale RFID deployments. In practical applications, our hardware support relies on the ImpinJ Speedway R420 reader, requiring no modifications like [38], compliant with the EPC Gen2 standard. The standard of EPC GEN2 [32] provides powerful tools and standardized solutions for item identification, tracking, and management. Leveraging previous successful applications [34,39] of commercially available RFID systems, we ensure the practical feasibility of our protocol.

## 7. Conclusions

In this work, we proposed an efficient early-breaking estimation and tree-splitting missing RFID tag identification (ETMTI) protocol to identify missing tags with the presence of unexpected unknown tags in large-scale RFID systems. ETMTI utilizes two novel strategies: the early-breaking estimation-based unknown tag deactivation (EBUD) method and the tree-splitting-based missing tag identification (TSMTI) method. Theoretical analysis yielded optimal parameters for both EBUD and TSMTI. Simulation results consistently demonstrate that ETMTI achieves a lower false negative rate and significantly reduces the time required for missing tag identification. Our research offers an efficient and reliable solution for real-world RFID systems. In future work, we plan to enhance the ETMTI protocol by implementing advanced collision reconciliation and compression techniques. Specifically, we will explore the integration of machine learning algorithms to intelligently reconcile collisions and optimize the identification process. Additionally, investigating novel data compression methods such as adaptive compression filters to further reduce the time cost. These targeted enhancements aim to significantly improve the overall efficiency and performance of the ETMTI protocol.

## Figures and Tables

**Table 1:** Effect of γ on the estimation process.

γ	1/2	1/4	1/8	1/16
** ϵ **	10.70%	16.54%	25.89%	30.18%
** $T_{est}$ **	55.37	29.06	16.8	9.6

**Table 2:** False negative rate of TSMTI when the unknown tag ratio varies.

$r_{ud}$	0.01	0.05	0.10	0.15	0.20
$\nabla_{fn}$	0.007	0.035	0.069	0.099	0.128

**Table 3:** Scenario Settings.

	Senarios	α	K	$r_{m}$	$r_{u}$
Phase I	$S_{11}$	0.95	[1000,5000]	0	0.1
	$S_{12}$	0.99	[1000,5000]	0	0.1
	$S_{13}$	0.95	3000	0	[0.1,1]
	$S_{14}$	0.99	3000	0	[0.1,1]
Phase II	$S_{21}$	-	[1000,5000]	0.3	0
	$S_{22}$	-	3000	[0.1,1]	0
Overall	$S_{31}$	0.95 or 0.99	[1000,5000]	0.3	0.1
	$S_{32}$	0.95 or 0.99	3000	[0.1,1]	0.1
	$S_{33}$	0.95 or 0.99	3000	0.3	[0.1,1]

## Data Availability

Data are contained within the article.
